# *Ralstonia mannitolilytica* infections in an oncologic day ward: description of a cluster among high-risk patients

**DOI:** 10.1186/s13756-017-0178-z

**Published:** 2017-02-07

**Authors:** Claudia Lucarelli, Enea Gino Di Domenico, Luigi Toma, Domenico Bracco, Grazia Prignano, Maria Fortunati, Lorella Pelagalli, Fabrizio Ensoli, Patrizio Pezzotti, Aurora García-Fernández, Annalisa Pantosti, Loredana Ingrosso

**Affiliations:** 1 Istituto Superiore di Sanità Viale Regina Elena, 299 00161 Rome, Italy; 2 European Program for Public Health Microbiology Training (EUPHEM), European Centre for Disease Prevention and Control, (ECDC), Stockholm, Sweden; 3 Istituto Nazionale Tumori Regina Elena, Istituto Dermatologico San Gallicano, Rome, Italy

**Keywords:** *Ralstonia mannitolilytica*, Outbreak, Central venous catheter, Oncologic patients, Carbapenem resistance

## Abstract

**Background:**

*Ralstonia spp*, an environmental microorganism, has been occasionally associated with healthcare infections. The aim of this study was to investigate an outbreak caused by *Ralstonia mannitolilytica* in oncology patients.

**Methods:**

Case definition: Oncology outpatients attending a day ward, with positive blood and/or central venous catheter (CVC) culture for *Ralstonia* spp from September 2013 – June 2014. We analysed medical records, procedures and environmental samples. *R. mannitolilytica* was identified by 16S rRNA sequencing, and typed by Pulsed Field Gel Electrophoresis (PFGE); resistance to carbapenemes was investigated by phenotypic and molecular methods.

**Results:**

The patients (*N* = 22) had different malignancies and received different therapy; all had a CVC and 16 patients presented chills and/or fever. *R. mannitolilytica* was isolated from both blood and CVC (*n* = 12) or only blood (*n* = 6) or CVC tips (*n* = 4). The isolates had indistinguishable PFGE profile, and showed resistance to carbapenems. All the isolates were negative for carbapenemase genes while phenotypic tests suggests the presence of an AmpC β-lactamase activity,responsible for carbapenem resistance. All patients had had CVC flushed with saline to keep the venous access pervious or before receiving chemotherapy at various times before the onset of symptoms. After the first four cases occurred, the multi-dose saline bottles used for CVC flushing were replaced with single-dose vials; environmental samples were negative for *R. mannitolilytica.*

**Conclusions:**

Although the source of *R. mannitolilytica* remains unidentified, CVC flushing with contaminated saline solution seems to be the most likely origin of *R. mannitolilytica* CVC colonization and subsequent infections. In order to prevent similar outbreaks we recommend removal of any CVC that is no longer necessary and the use of single-dose solutions for any parenteral treatment of oncology patients.

## Background

The genus *Ralstonia* comprises a group of non-fermentative, Gram-negative bacteria (NFGN) found in moist environments, such as water, soil and plants [1]. Three Ralstonia species, *Ralstonia pickettii*, *Ralstonia insidiosa* and *Ralstonia mannitolilytica*, formerly designated *Burkholderia pickettii*, *Burkholderia solanacearum* and *Pseudomonas thomasii,* respectively, have been recognized as opportunistic human pathogens [1]. Their relevance has been currently re-evaluated because of their ability to survive in different types of disinfectants and to pass through 0.2-μm filters that are used to sterilize solutions [1, 2].

*Ralstonia* spp. is reported as a causative agent of bacteremia [3, 4], meningitis [5, 6], and sepsis [7, 8] in immunocompromised patients and of central venous catheter (CVC)-associated bacteremia in oncology patients [1–4, 9]. Several hospital outbreaks have been described that were associated with contaminated solutions, including water for injection, saline solutions, disinfectants and antiseptics [1]. Multidrug resistance in NFGN is widely reported in the literature [10, 11] and is causing increasing concern because such bacteria may have a role not only as human pathogens but also as potential reservoirs of resistance genes, particularly when they are found in hospital settings. Several studies have described resistance to fluoroquinolones, 3^rd^ generation cephalosporin and carbapenems [1] in isolates belonging to all the three *Ralstonia* species.

*R. pickettii* is the *Ralstonia* species most frequently reported in the literature while only a limited number of infections are attributed to *R. insidiosa* and *R. mannitolilytica* [1]. The clinical importance of these two species is probably underestimated because their biochemical patterns are similar to that of *R. pickettii,* making it impossible their distinction based on conventional microbiological tests only [1, 4, 12, 13].

Here we describe an outbreak caused by *R. mannitolilytica* in patients attending a day ward unit in an oncology hospital in Rome occurred from September 2013 – June 2014.

## Methods

### Hospital setting

The Istituto Nazionale Tumori Regina Elena – Istituto Dermatologico San Gallicano is a 215-bed hospital located in Rome with approximately 6,700 inpatient admissions and 900,000 outpatients visits per year. The hospital has two oncology day wards (DW-A and DW-B) located in different buildings, each consisting of a single therapy room with 20 bays for chemotherapy infusion and attended by an average of 65 patients/day. Patients are assigned to either ward while the infusion bays are not pre-assigned neither a register for the allocation of patients to bays is in place. One of the units (DW-A) was affected by the outbreak and was the object of the epidemiological investigation. From September 2013 – June 2014, 2485 patients attended DW-A for a total of 20,177 day-hospital visits.

### Epidemiological investigation

We defined a case as a patient attending DW-A from September 2013 – June 2014 that had a blood culture and/or a CVC tip culture positive for *Ralstonia* spp. with or without symptoms (chills and/or fever). Two of the authors (CL, LI) assisted by an infection control nurse (MF) reviewed all the medical records of the cases to identify any common medical procedure or any occurrence that might have posed a risk for acquiring *Ralstonia* spp. infection.

### Environmental sampling

Environmental sampling started on 17 October 2013, immediately after *Ralstonia* spp. had been isolated from the blood culture of the third case. Laboratory technicians collected the samples using commercially available sterile swabs (COPAN Eswabs, Brescia, Italy), following existing departmental guidelines, in the therapy room from furniture and electronic devices (*N* = 8), and in the drug preparation room (*N* = 8) from personal computer, telephone, fax, medicine cabinet, medicine trolley. Swabs were cultured in Tryptic Soy Broth, incubated for 48 h at 37°C, and plated on chocolate agar and blood agar. Samples were also obtained from liquid soaps (*N* = 10), their dispensers (*N* = 8) and chlorhexidine (*N* = 4) in use in DW-A. In addition distilled water and sterile water used for injection were cultured as described in Moreira et al. [14].

### Microbiological methods and typing of isolates

Blood samples were taken through CVC in all patients*. R. mannitolilytica* was isolated from signal-positive blood culture bottles (BacT/ALERT BioMérieux, Florence, Italy) using conventional methods. Sixteen CVC tips were cultured according to Zhang et al. [15].

Identification of the isolates was obtained by VITEK 2 system (BioMérieux, Florence, Italy). Identification at the species level was obtained by amplification of the 16S rDNA gene followed by double-strand sequencing [16].

To investigate the relatedness of *Ralstonia* isolates, PFGE was performed on all isolates obtained from blood cultures and CVC tip cultures, following digestion of genomic DNA by the restriction enzyme *Spe*I (New Englands Biolabs, Ipswich, MA), according to the CDC protocol (available at https://www.cdc.gov/pulsenet/pdf/ecoli-shigella-salmonella-pfge-protocol-508c.pdf) with addition of 50 μM thiourea in the agarose gel and in the electrophoresis running buffer. The following running conditions were used: 120 costant angle at 6V/cm, with pulse time 20h 1s-40s, 4h 30s-60s. *Salmonella* Braenderup H9812 was used as reference for molecular size.

### Antibiotic susceptibility of the isolates

Susceptibility testing of the isolates was performed by the disk diffusion method according to the EUCAST guidelines (available at http://www.eucast.org/clinical_breakpoints/). The antimicrobials tested were: ceftazidime, meropenem, ciprofloxacin, gentamicin, amikacin, and piperacillin/tazobactam (Becton Dickinson, Milan, Italy). Antimicrobial susceptibility to piperacillin/tazobactam was confirmed by Etest (BioMérieux, Florence, Italy). As there are no EUCAST susceptibility breakpoints available for *Ralstonia* spp., the results were interpreted using the EUCAST criteria for *Pseudomonas* spp. *Pseudomonas aeruginosa* ATCC 27853 was included as control.

### Identification of determinants of carbapenem-resistance

Four outbreak isolates (A, E, L, S) were randomly selected to perform the phenotypic and molecular tests. Identification of resistance mechanisms for carbapenems was performed by the agar tablet/disc diffusion method (KPC/MBL and OXA-48 Confirm Kit, ROSCO Diagnostica A/S, Taastrup, Denmark). In addition, PCR assays were performed for the identification of the chromosomal genes *bla﻿*_OXA-443_ and *bla*_OXA-444_, previously described in a carbapenem-resistant *R. mannitolilytica* strain [17]. For amplification of the two genes, two couple of primers were designed: OXA-443 Fw 5’-ATGACGAAACTCCGCCA-3’/OXA-443 Rv 5’-AGGTGGGCTCGATCTTG-3’ and OXA-444 Fw 5’-ATGTTCTCTCGTTGGTC-3’/OXA-444 Rv 5’- TGCGGGTCGGACGGAGA -3’. The presence of other carbapenem-resistance genes was investigated by multiplex PCR assay with primers designed to amplify the following 11 genes: *bla*_IMP_, *bla*_VIM_, *bla*_NDM_, *bla*_SPM_, *bla*_AIM_, *bla*_DIM_, *bla*_GIM_, *bla*_SIM_, *bla*_KPC_, *bla*_BIC_, and *bla*_OXA-48_, accordingly to Poirel et al. [18].

### Control strains

All the experiments were performed using two controls strains: *R. mannitolilytica* BK931 [2] and *R. mannitolilytica* ATCC BAA-716 (LMG 6866) [13].

## Results

### Epidemiological and microbiological investigation

**Fig. 1:**
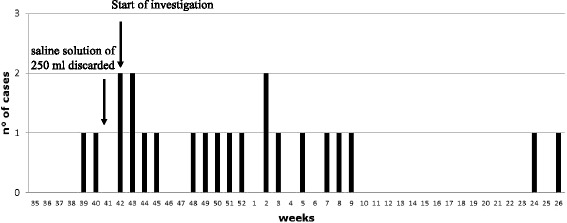
Epidemic curve of cases of *R. mannitolilytica* in oncology patients attending the day hospital ward A, September 2013-June 2014

According to case definition, we identified 22 patients (attack rate 0,88%), 13 males and 9 females, age range 30–84 years old (median age 66), attending DW-A from 24 September 2013 – 23 June 2014 (Fig. 1, Table 1). In particular, 12 patients had *Ralstonia* spp. positive cultures from both blood and the CVC tip; four patients didn’t have blood culture performed, but because of the clinical symptoms their catheters were removed and cultured and were found positive for *Ralstonia* spp*.*; other six patients had a positive blood culture but the CVC tips were not tested.

**Table 1:** Data of the 22 cases (A-V) of *R. mannitolilytica* outbreak, September 2013-June 2014, oncology hospital in Rome

Patient	Sex	Age	Type of CVC	Date of implantation	Date of diagnosis	Blood culture	Tip CVC culture
A	F	62	PICC	08/08/2013	24/09/2013	+	ND
B	M	56	Port	07/06/2011	02/10/2013	+	ND
C	M	84	Port	18/09/2012	16/10/2013	+	+
D	M	66	Port	02/04/2013	16/10/2013	+	+
E	F	66	Port	13/12/2011	24/10/2013	+	+
F	F	62	PICC	10/06/2013	24/10/2013	+	ND
G	F	73	Port	31/05/2011	28/10/2013	+	+
H	F	65	Port	NA	07/11/2013	+	+
I	M	67	Port	13/12/2011	29/11/2013	+	+
J	M	56	Port	NA	03/12/2013	+	+
K	F	30	Port	25/09/2012	14/12/2013	+	+
L	F	65	Port	NA	20/12/2013	ND	+
M	M	62	Port	10/10/2010	23/12/2013	+	+
N	F	72	Port	NA	07/01/2014	+	+
O	M	77	Port	NA	09/01/2014	+	ND
P	F	67	Port	NA	14/01/2014	ND	+
Q	M	80	Port	15/04/2009	30/01/2014	+	ND
R	F	80	Port	NA	14/02/2014	+	ND
S	F	63	Port	NA	21/02/2014	+	+
T	M	70	Port	NA	27/02/2014	+	+
U	M	61	Port	31/01/2013	12/06/2014	ND	+
V	M	61	Port	05/12/2011	23/06/2014	ND	+

*NA*: not available; *ND*: not determined

Analysis of the medical records revealed that the patients had different types of CVC, in particular 20 had a Port, while two had a PICC (percutaneous introduction central catheter). We were able to retrieve the insertion dates of the devices for 13 out of 22 patients: they were inserted from more than 4 years to less than 2 months before the onset of symptoms (Table 1). The patients had different types of solid cancer, they underwent different therapeutic protocols and attended DW-A in different days. The only common procedure among the patients that we were able to identify was a maintenance procedure, which consisted in flushing saline solution through the CVC, after disinfection with 2% chlorhexidine gluconate in 70% isopropyl alcohol. Such procedure was performed either before the patient received chemotherapy or as a periodic standard procedure to keep the patient’s venous access pervious. Sixteen patients reported fever and/or chills while receiving chemotherapy through CVC or within two hours after the CVC flushing procedure. No information was available for the remaining six patients. No patient had signs of skin infection at the Port site or at the PICC insertion site. Empirical antibiotic therapy (ciprofloxacin) was deemed necessary for one patient only. All patients had their CVCs removed and this led to resolution of symptoms in all cases.

The review of the current medical practices of DW-A revealed that, between August 2013 and September 2013, five bottles of saline solution of 250 ml were used for CVC flushing. This type of bottle was in use only in DW-A, and not in DW-B, and usually remained in use for two days from opening. All the cases attended the DW-A for CVC flushing when the 250 ml bottles of saline solution were in use. After the occurrence of the first three cases, the hospital committee for control of healthcare-associated infection gave indications to perform environmental cultures and room disinfection (mid October). However, since the 250 ml bottles of saline solution had been already discarded and replaced with single-dose vials, they were unavailable for microbiological testing when the investigation was started. All the environmental samples and samples from chlorhexidine, distilled water and sterile water were negative for the presence of *Ralstonia* spp.

### Characterization of the pathogen

**Fig. 2:**
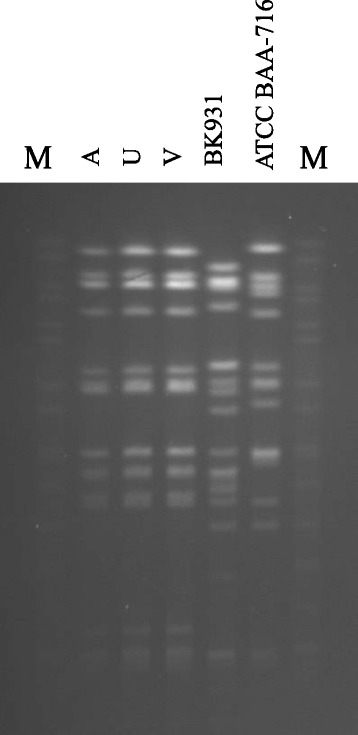
Pulsed-field gel electrophoresis (PFGE) of *R. mannitolilytica* strains. M: Molecular weight standard, *Salmonella* Braenderup H9812. A: strain isolated from blood culture. U, V: strains isolated from CVC. These strains are representative of the entire sample. BK931, ATCC BAA-716: control strains

*Ralstonia* spp. had never been isolated from any patients in the hospital both before and after the outbreak. By routine biochemical tests the isolates from blood and CVC tips were identified as *R. pickettii*. However, 16S rDNA sequencing showed that the isolates were in fact *R. mannitolilytica*. In addition, all the isolates shared an indistinguishable PFGE profile (Fig. 2) while the two control strains (ATCC BAA-716 and BK931), showed profiles that were distinct from those of the outbreak strains. The outbreak strains had the same multi-drug resistance profile: they were resistant to ceftazidime, meropenem, ciprofloxacin, gentamicin, and amikacin but were susceptible to piperacillin/tazobactam (MIC ≤16 mg/L). Except for susceptibility to ciprofloxacin, the two control strains had the same resistance pattern.

Regarding resistance to carbapenems the strains were negative for the presence of the 11 carbapenemase genes screened by multiplex PCR. All the isolates tested, including the two controls, were positive by PCR for the *bla*_OXA-443_ and *bla*_OXA-444_ genes. Phenotypic tests performed to assess the mechanism of carbapenem resistance showed that cloxacillin was able to abolish meropenem resistance, thus suggesting the presence of an AmpC β-lactamase activity.

## Discussion

The microbiological and epidemiological investigation of this outbreak did not detect the source of the contamination, however the molecular typing of the pathogen strongly supports the hypothesis of a common source of contamination. Failure to identify the culprit is probably due to the fact that when the investigation was started samples from disinfectants, antiseptics and saline solutions used at the beginning of the outbreak were not available for microbiological investigation. In fact, disinfectants, antiseptics and multi-dose bottles of saline solution have been described in the literature as one of the main sources for *Ralstonia *spp. contamination [1], therefore their prompt removal from DW-A immediately after the beginning of the outbreak was the most understandable precaution to adopt from the patients’ safety viewpoint. However, in spite of the multi-dose bottles of saline solution removal, the outbreak lasted 9 months. Indeed, the revision of the medical records showed that CVC flushing with saline solution was a common procedure adopted with all the patients and that, at the beginning of the outbreak, multi-dose bottles of saline solution were in use in DW-A. Thus it is very likely that patients were exposed to one or more contaminated bottle(s) of saline. The majority of the patient developed clinical symptoms immediately thereafter, while others likely had a CVC colonization by *R. mannitolilytica*. Any subsequent procedure through CVC, chemotherapy or new saline flushing, may have caused detachment and dissemination of *R. mannitolilytica* from CVC, causing fever and chills. This might have happened even after several weeks from CVC colonization, possibly accounting for the protracted duration of the outbreak. A similar finding have been reported by Raveh et al. [19] in which, a patient with a CVC developed fever and chills and his blood culture was positive for *R. pickettii*. The patient was treated with antibiotic therapy and the CVC was left in place. However 15 months later the patient developed another bloodstream infection with the same organism likely colonizing CVC.

The ability to grow as biofilm on abiotic surfaces plays an important role in colonization of hospital equipment and indwelling medical devices such as CVCs [20]. Ability to form biofilm has been described in different *Ralstonia* species including also *R. mannitolylitica* [21–24]. It is conceivable that biofilm formation might have played a role for these strains to allow adherence to CVCs and subsequent dissemination in the host, following the flushing procedures of the device.

The prolonged duration of this outbreak is not unprecedented for this pathogen. Daxboeck et al. [4] reported isolation of *R. mannitolilytica* in 30 patients attending 15 different wards between February 2002 and March 2004; in this study also the source was not identified. Other outbreaks have been reported in pediatric patients, associated with oxygen delivery devices [25, 26] and in oncology patients [1, 2]. In the outbreak described by Gröbner et al. [2], which lasted 11 weeks, the source was not identified but it was hypothesized that a contaminated solution administered through CVC could have been the culprit.

It is important to underline that the misuse of multidose vials, as in our outbreak, has been frequently reported as a cause of numerous outbreaks [27–30] in different European countries as well as in USA where the CDC is aware of at least 49 outbreaks occurred in 13 years [31]. Therefore the correct use of single dose vials, for a single patient, is an effective and important way to prevent outbreaks as reported by specific guidelines [32].

*R. mannitolilytica* is frequently recognized as a multiresistant microorganism [4, 33–35] but limited data are available about carbapenem-resistance. To the best of our knowledge, only a limited number of studies so far, have tested susceptibility to this drug: in four studies, all the strains tested were resistant (five in one study and one strain each in the other three) [2, 17, 33] while Daxboeck et al. [4] reported carbapenem-resistance in 12 strains out of 30. In our isolates no carbapenemase genes were detected, but they were positive for the chromosomal genes *bla*_OXA-443_ and *bla*_OXA-444_, that have been previously described in one *R. mannitolilytica* carbapenem-resistant strain [17] and that bear close similarity to the *bla*_OXA-22_ and *bla*_OXA-60_ genes found in *R. pickettii* [36]. *bla*_OXA-22_ and *bla*_OXA-60_ code for two oxacillinase, a narrow spectrum oxacillinase and an inducible carbapenemase, respectively, although none of them was found to be responsible for carbapenem resistance in *R. pickettii* [36, 37]. In addition, the cloxacillin test suggested that the *R. mannitolilytica* isolates were positive for the presence of AmpC β-lactamases. These enzymes are clinically important because they may confer resistance to a wide variety of β-lactam drugs, narrow-, expanded-, and broad-spectrum cephalosporins, β-lactam-β-lactamase-inhibitor drugs combinations, as well as to aztreonam and carbapenems in case of association with altered porins and/ or efflux mechanisms [38]. Taken together, our data suggest that meropenem resistance was likely due to overproduction of AmpC β-lactamase, possibly in synergy with a second mechanism (e.g. decreased production of the porin channel and/or activation of efflux systems). Recently, a serin-hydrolase class C family β-lactamase has been identified in *R. mannitolilytica* (GenBank accession number: WP_045219476) [15], therefore production of this enzyme may contribute to carbapenem resistance.

Although *R. mannitolilytica* did not cause life-threatening infections in this outbreak as well as in previously reported outbreaks [2, 26], the increased detection of *Ralstonia* spp. in healthcare settings coupled with the emergence of multi-resistant strains of *R. mannitolilytica,* represent a reason of concern, particularly in case of vulnerable patients which may require an antimicrobial therapy.

## Conclusion

We reported the first outbreak due to *R. mannitolilytica* in oncology patients bearing CVC in Italy. Although the source of the outbreak could not be readily identified, the investigation suggested that contaminated saline solution used for CVC flushing may have been the source of the outbreak. In order to prevent possible infections we recommend the removal of any CVC that is not longer necessary and the use of single dose solutions for any parenteral treatment of cancer patients.

## Abbreviations

CVC: Central venous catheter
DW-A: Day ward A
DW-B: Day ward B
MIC: Minimal inhibitory concentration
NFGN: Non-fermentative gram-negative bacteria
PCR: Polymerase chain reaction
PFGE: Pulsed field gel electrophoresis
PICC: Percutaneous introduction central catheter
